# Correcting the predictive validity of a selection test for the effect of indirect range restriction

**DOI:** 10.1186/s12909-017-1070-5

**Published:** 2017-12-11

**Authors:** Stefan Zimmermann, Dietrich Klusmann, Wolfgang Hampe

**Affiliations:** Department of Biochemistry and Molecular Cell Biology, University Medical Center Hamburg-Eppendorf, Martinistraße 52, D-20246 Hamburg, Germany

**Keywords:** Predictive validity, Student selection, Range restriction, Suppression, Mice, EM

## Abstract

**Background:**

The validity of selection tests is underestimated if it is determined by simply calculating the predictor-outcome correlation found in the admitted group. This correlation is usually attenuated by two factors: (1) the combination of selection variables which can compensate for each other and (2) range restriction in predictor and outcome due to the absence of outcome measures for rejected applicants.

**Methods:**

Here we demonstrate the logic of these artifacts in a situation typical for student selection tests and compare four different methods for their correction: two formulas for the correction of direct and indirect range restriction, expectation maximization algorithm (EM) and multiple imputation by chained equations (MICE). First we show with simulated data how a realistic estimation of predictive validity could be achieved; second we apply the same methods to empirical data from one medical school.

**Results:**

The results of the four methods are very similar except for the direct range restriction formula which underestimated validity.

**Conclusion:**

For practical purposes Thorndike’s case C formula is a relatively straightforward solution to the range restriction problem, provided distributional assumptions are met. With EM and MICE more precision is obtained when distributional requirements are not met, but access to a sophisticated statistical package such as R is needed. The use of true score correlation has its own problems and does not seem to provide a better correction than other methods.

## The problem

If the predictive validity of an admission test is low, its employment is hard to justify. Therefore a trustworthy estimation of predictive validity is needed. The predictive validity of a test is defined as the correlation of its scores with an outcome criterion. However in many test situations this correlation is not directly calculable. Here we consider two complications which lead to an underestimation of predictive validity:

1. The selection decision is based on multiple criteria, which can compensate for each other. This generates a negative correlation between predictors which attenuates the predictor-outcome correlation for each of them.

2. As outcome data are not available for rejected applicants the range of predictors as well as of the criterion variable is restricted and this attenuates the predictor-outcome-correlation.

Both problems are often ignored or treated superficially. This seems to reflect a lack of communication between psychometric statisticians and applied researchers, who may feel deterred by the multitude of methods offered and their seemingly inscrutable subtleties [1]. In this article we will describe the problems of calculating predictive validity in an illustrative scenario using artificial data, then demonstrate methods for their solution, and finally apply these methods to real data from an entrance test for medical school which is used in combination with high school GPA (hGPA) in Germany. We hope that applied researchers will find this account helpful for their understanding of artifact correction in validity estimation and finally adopt one of the proposed solutions in their own research.

## Illustrative scenario

Let’s imagine a situation typical for student selection: Two selection variables X_1_ (for example an entrance test) and X_2_ (for example hGPA) are combined into a selection score Z = X_1_ + X_2_. Applicants are admitted according to their rank order on Z. In the following, we present artificial data generated to illustrate the scenario. We use the software R with the package MASS [2] to draw data points for X_1_, X_2_, and Y from a standard normal random distribution (mean = 0, sd = 1) restricted to yield the following correlations: ${\uprho}_{y{x}_1\mid a}=.60$, ${\uprho}_{y{x}_2\mid a}=.20$, and ${\uprho}_{x_1{x}_2\mid a}=.00$, (*N* = 1000 applicants).

Following the terminology of McManus [3] the total sample of all test takers is called “applicants”, and the selected subgroup (here 20% of applicants) is called “incumbents” because this group usually is entitled to something, e.g. a study place. In this article we denote applicants with *a*, incumbents with *i*. Examples: ${r}_{yx_1\mid a}$ is the correlation between the outcome Y (study success) and the predictor X_1_ (test) in the applicants, ${r}_{yx_{1\bullet 2}\mid i}$ is the correlation between the outcome Y and the predictor X_1_ in the incumbents after the linear effect of X_2_ (hGPA) has been removed from X_1_. X_1_ is the predictor of interest - we want to know how well the test performs in predicting the outcome Y independent of X_2_. While X_1_ and X_2_ are measured in all applicants, the outcome variable Y is only measured in the incumbents. Thus, ${r}_{y{x}_1}$ is only defined in the incumbents (${r}_{y{x}_1\mid i}$), as we do not have data about the outcome from the rejected applicants. Still we want to know the relationship between X_1_ and Y in the unrestricted sample (${r}_{y{x}_1\mid a}$).

### Compensatory selection

**Fig. 1:**
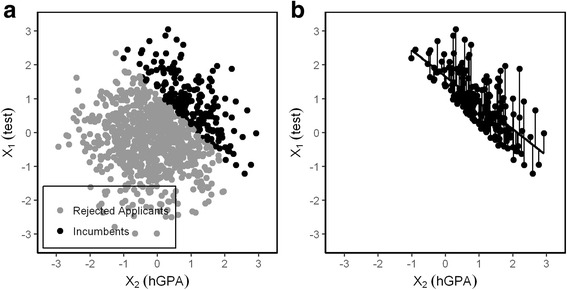
**a** Scattergram of X_1_ and X_2_. 20% of 1000 applicants are selected by the sum of X_1_ and X_2_; the circular cloud representing all applicants is divided by a diagonal line that separates the top right area from the bottom left area. **b** This generates a negative correlation between X_1_ and X_2_ in the incumbents $\left({r}_{x_1{x}_2\mid i}=-0.71\right)$. Residuals of X_1_ after the linear effect of X_2_ is removed. They are expressed as deviations from the regression line: The residuum of X_1_ when the influence of X_2_ is removed is the observed X_1_ value minus the expected value of the regression X_1_ on X_2_

Here we demonstrate why compensatory selection is a factor that attenuates the estimation of predictive validity. If two measures are combined into a sum that is used for the selection decision, e.g. a test score for natural science knowledge (X_1_), and high school grade point average (hGPA) (X_2_) a low test score might be compensated by a high hGPA and vice versa. Only if both measures are low an applicant will be rejected which creates a negative correlation ${r}_{x_1{x}_2\mid i}$ in the incumbents (Fig. 1).

The negative correlation has repercussions for predictive validity: the negative correlation ${r}_{x_1{x}_2\mid i}$generated by the selection procedure diminishes the correlations of each of the two predictors with the outcome Y - a suppressor effect occurs. A suppressor variable is a variable, which increases the predictive validity of another variable (or a set of variables) by its inclusion in a regression equation. It improves predictability by purging some irrelevant variance from other predictors [4].

Reciprocal suppression is the case we are concerned with: Two negatively correlated predictors act as suppressors for each other. Reciprocal supression has been thoroughly analyzed by Lutz [5] and Conger [6]. In our case the origin of the negative correlation is clear: It is the compensatory selection rule that has eliminated all applicants with low values in both variables from the sample. The correlation of X_1_ and X_2_ in the unrestricted sample of applicants is almost zero (${r}_{x_1{x}_2\mid a}=0.01$), but in the restricted sample of incumbents it is negative (${r}_{x_1{x}_2\mid i}=-0.71$) because whenever the value of one variable is low the value of the other variable must be moderately high at least - otherwise the applicant would have been rejected. This has implications for the relation of both predictors X_1_ and X_2_ to the outcome Y within the incumbents.

**Fig. 2:**
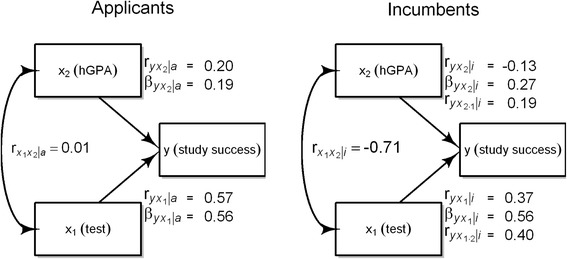
Relations between X_1_, X_2_, and Y in applicants and incumbents for 20% selection rate. ${r}_{y{x}_1}$: first order correlation, ${\beta}_{y{x}_1}$: beta coefficient, ${r}_{yx_{1\bullet 2}}$: semipartial correlation

An applicant with a low hGPA (X_2_) can still be accepted if her entrance test result (X_1_) is superior. A low hGPA would predict less study success (Y), whereas a high test results would predict more study success. Thus the negative relation between the test score and hGPA would mask the predictive power of the test. This also goes vice versa, if entrance test and hGPA change places. Multiple regression removes the effect of reciprocal suppression as it shows the independent contribution of each predictor after controlling for all other predictors (similar to the calculation of the residuals in Fig. 1). The beta coefficients of the two predictors exceed their first order correlations with the criterion (Fig. 2). However beta coefficients are not correlations, but weights which determine how much Y is expected to respond if X_1_ changes one unit of a standard deviation. This is especially helpful if we want to compare the influence of multiple variables that possess different measurement units [7]. As we need to assess the validity of X_1_ as a unique predictor the semipartial correlation is more suitable because it represents the correlation of the residuals of X_1_ after the linear effect of X_2_ has been taken out [8]. The semipartial coefficient has the same numerator as the standardized regression coefficient β but a slightly different denominator, $\sqrt{1-{r}_{x_1{x}_2}^2}$ instead of the square of this term, $1-{r}_{x_1{x}_2}^2$. While the semipartial coefficient is bound between −1 and +1, the standardized regression coefficient is not. The semipartial correlations ${r}_{yx_{1\bullet 2}\mid i}$ and ${r}_{yx_{1\bullet 2}\mid i}$ not only correct for the compensatory selection procedure but for any effect X_2_ may have on X_1_ and vice versa. So our first step to the correction of the empirical predictor-outcome correlation is to exclude the effect of reciprocal suppression by computing the semipartial correlation. Instead of ${r}_{y{x}_1\mid i}=.37$we obtain ${r}_{yx_{1\bullet 2}\mid i}=.40$ as an estimation of predictive validity corrected for compensatory selection. This still deviates from the true correlation which we know because we work with artificial data: ${r}_{y{x}_1\mid a}=0.57.$ The difference between ${r}_{yx_{1\bullet 2}\mid i}=.40$ and ${r}_{y{x}_1\mid a}=0.57$ must be attributed to the effect of range restriction.

### Range restriction

Correlations derived from the subgroup of selected applicants are often simply reported as indicators of predictive validity without correcting for the effects of multiple selection variables and range restriction [9]. This error has been observed more than half a century before: Cyril Burt exposed the “time-honoured fallacy”, of, “judging the efficiency of [an] examination as a mean of selection by stating its efficiency as a means of predicting the order of merit within the selected group”, [10] p. 2. This is an instance of the fallacy of composition, which arises whenever one infers that something is true of the whole from the fact that it is true of some part of the whole, in this case the correlation coefficient for the whole group of applicants taken erroneously as identical to the correlation coefficient for the admitted applicants. McManus deplores the widespread tolerance for this fallacy and the consequential misinterpretation of findings: “Even in prestigious journals a naïve interpretation can be made that selection measures, such as A-level grades, are actually of little value” [3], p. 4.

Many studies in the field of predictive validity do not correct for range restriction. For example a recent article on the validity of the UKCAT reports uncorrected predictor-outcome correlations as validity coefficients [11]. Such coefficients are typically very low and lead to disappointing conclusions about the predictive validity of a test or attempts to shrug off such conclusions. As attenuation due to range restriction is inversely associated with the selection rate its omission is particularly detrimental in highly selective admission procedures as in selection for medical school. Artificially low validity coefficients are not in the interest of most research teams, so why does this happen? Perhaps some researchers do not know that range restriction is a problem, or they know, but feel uneasy about remedial action because it feels like arbitrarily jacking up a correlation coefficient or “armchair magnification” [10], p. 13. However corrected correlations are less biased than uncorrected correlations, even when assumptions are not met fully [12, 13]. Correction for range restriction is recommended by the Society for Industrial and Organizational Psychology as a matter of routine [14]*.*

How can we estimate the predictive validity for all applicants if we have no outcome data for a large part of the sample? A multitude of methods have been developed to achieve this feat in a variety of testing situations [1]. The menu of suitable methods comprises multiple correction formulas using different sets of assumptions as well as maximum likelihood methods of missing value imputations such as Bayesian Monte Carlo methods [15–17].

**Fig. 3:**
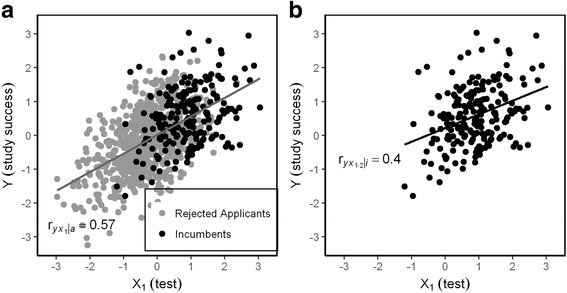
The correlation in the full sample of applicants (**a**) is larger than the correlation in the incumbents (**b**) due to range restriction: The variances of X_1_, X_2_ and Y are restricted

Imagine an ellipsoid of points in two-dimensional space representing the unknown predictor/outcome correlation in the total sample (Fig. 3a). If the lower part of the ellipsoid is removed by a selection rule, only the black points at the right side remain and this cloud corresponds to a correlation coefficient much lower than the one that corresponds to the full ellipsoid (Fig. 3b). The correlation in the full ellipsoid might be recovered, if its shape continued from the known black points to the right to the unknown gray points to the left in an orderly way.

We will consider four methods for correcting the effect of range restriction:

1. Thorndike’s case A formula for selection with a single variable
2. Thorndike’s case C formula for indirect selection
3. Expectation Maximization (EM)
4. Multiple imputation if missing values by chained equations (MICE)

Methods 2–4 handle indirect range restriction, so a correction for the effect of reciprocal suppression due to compensatory selection is not necessary. We included this analysis for clarification, not with the intention to use the semipartial correlation ${r}_{yx_{1\bullet 2}\mid i}$, albeit with one exception: In Thorndike’s case A formula we replace X_1_ by the residual X_1•2_, thus ${r}_{y{x}_1\mid i}$ by ${r}_{yx_{1\bullet 2}\mid i}$in order to account for the suppression caused by X_2_.

*Thorndike’s case A formula* [18] is widely used for the correction of range restriction. This formula estimates the correlation in the non-restricted sample (non-restricted correlation), if three coefficients are given: (1) the restricted correlation ${r}_{y{x}_1\mid i}$ (2) the restricted standard deviation ${\mathrm{SD}}_{x_1\mid i}$ and (3) the non-restricted variance ${\mathrm{SD}}_{x_1\mid a}.$ It works in a situation where the performance of a single predictor X_1_ is evaluated with an outcome Y.

Thorndike’s case A formula depends on two assumptions [19]:

1. Linearity. $$ {b}_a=\frac{r_{y{x}_1\mid a}}{SD_{x_1\mid a}^2}={b}_i=\frac{r_{y{x}_1\mid i}}{SD_{x_1\mid i}^2} $$
2. Homoscedasticity. The error variance *e* in Y is the difference between the observed Y value and the expected value in Y that the regression model implies. Homoscedasticity requires the error variance *e* to be equal for the applicants and the incumbents:

$$ {SD}_{e\mid a}^2={SD}_{y\mid a}^2\ \left(1-{r}_{y{x}_1\mid a}^2\right)={SD}_{e\mid i}^2={SD}_{y\mid i}^2\ \left(1-{r}_{y{x}_1\mid i}^2\right) $$

Let $u=\frac{{\mathrm{SD}}_{x_1\mid a}}{{\mathrm{SD}}_{x_1\mid i}}$ be the ratio of unrestricted to restricted standard deviation.

Then the estimation of the unrestricted correlation ${r}_{y{x}_1\mid a}$ follows unambiguously from assumptions (a) and (b). After some transformation (see [20]) the result is: ${r}_{y{x}_1\mid a}=\frac{u{r}_{y{x}_1\mid i}}{\sqrt{\left(1-{r}_{y{x}_1\mid i}^2+u{r}_{y{x}_1\mid i}^2\right)}}$

If the error variance in Y is equal at any level of X_1_ (homoscedasticity), then it does not matter at which location on the X-axis the distribution has been cut by the selection rule as long as the cut is clean and not rendered fuzzy by the involvement of a third variable such as X_2_.

In the empirical literature Thorndike’s case A formula is often used even when selection is also guided by other variables than X_1_ (indirect selection). Schmidt and Hunter [21] demonstrated that this regularly leads to substantial underestimation of the unrestricted correlation coefficient. We include Case A in our list of correction methods to demonstrate the magnitude of underestimation caused by ignoring indirect selection.

*2. Thorndike’s case C formula for indirect selection.* In our simulation we have a second selection variable X_2_, which is not envisaged by Thorndike’s case A formula. If multiple variables are used for selection, a compound selection variable Z is formed as a function of X_1_, X_2_, … X_k_, e.g. Z = X_1_+ X_2_ + … + X_k_ Thorndike’s case C formula incorporates such indirect (sometimes called incidental) selection based on Z, of which the variable of interest, X_1_, only is a part [22]:

$$ {r}_{y{x}_1\mid a}=\frac{\left({r}_{y{x}_1\mid i}^2+\left(u-1\right){r}_{y{x}_1\mid i}\right)}{\sqrt{1+\left(u-1\right)\ {r}_{y{x}_1\mid i}^2\ }\ \sqrt{1+\left(u-1\right)\ {r}_{z{x}_1\mid i}^2\ }} $$

*The EM algorithm* computes maximum likelihood estimates from incomplete data [23]. It fills in missing values (in our case the missing Y-values of rejected applicants) with their expectations conditional to a set of currently assumed parameter values (Expectation-step), then reestimates these parameters (in our case ${\widehat{r}}_{y{x}_1\mid a}$) and repeats the process until the estimates exhibit no important change (Maximization-step). An important assumption for an unbiased estimation of the EM algorithm concerns the mechanism that leads to the missing values. EM as well as MICE as a multiple imputation algorithm require data to be missing at random (MAR): if a variable is missing, it should not depend on its value itself but on other variables that could be observed and that are included in the model [24]. In a study which investigated the reconstruction of validity coefficients in the context of driving learners, the authors used a convincing research design. For the purpose of the study, driving learners who failed the theoretical test were also admitted for the practical driving test. They could show that EM performed well in predicting the true validity from an artificial restricted dataset [17]. In our analysis, we are using the EM algorithm as implemented in the package Selection for R [25].

*4. The MICE algorithm* is the most recent and according to its advocates the most accurate approach [15]. The acronym stands for Multiple Imputation by Chained Equations A multiple imputation analysis consists of three distinct steps: the imputation phase, the analysis phase, and the pooling phase. In the imputation phase a number *m* of complete datasets are created which contain different plausible estimates of the missing values, but identical values for the observed data. In the analysis phase, each complete dataset is analyzed with conventional statistical methods. In the pooling phase the *m* parameter estimates are pooled into a single set of parameter estimates by calculating simply the arithmetic average of the *m* estimates from the analysis phase. Via multiple imputations the variance in the variables with missing values is handled more realistically than in EM. MICE is a Bayesian type of estimation. In Bayesian statistics, a prior distribution of parameter values is modified in the light of new evidence to obtain a posterior distribution of improved estimates. MICE uses Markov chain Monte Carlo (MCMC) methods to find the posterior distribution of the parameters. MCMC algorithms need a large number of steps and therefore are CPU-intensive. Only with the recent advent of powerful PCs such methods have become widely available for applied research. We use the package MICE for R [26].

## Study 1: Monte Carlo simulation

The data shown to illustrate the issue of range restriction were used in a Monte Carlo simulation. We rerun the illustrative scenario (${\uprho}_{y{x}_1\mid a}=.60$, ${\uprho}_{y{x}_2\mid a}=.20$, and ${\uprho}_{x_1{x}_2\mid a}=.00$, *N* = 1000 applicants) 1000 times and varied the student selection ratio (10%, 20% and 30%) that leads to missing data on Y for the rejected applicants. As we know ${r}_{y{x}_1\mid a}$ from the data generation process we can test the precision of methods promising to infer ${r}_{y{x}_1\mid a}$, from ${r}_{y{x}_1\mid i}$ . The repetition of the data generation process and the application of the four range correction methods allow us to determine a point estimate and the variability for each correction method. The unrestricted correlation estimated by one of the four methods is denoted by ${\widehat{r}}_{yx_1\mid a}$. The difference between *r* and $\widehat{r}$ is the error of estimation for the unrestricted correlation. We use this indicator to assess the precision of the four correction methods. A value of 0.0 means that the unrestricted correlation coefficient could be perfectly recovered based on the data of the incumbents.

### Results

**Fig. 4:**
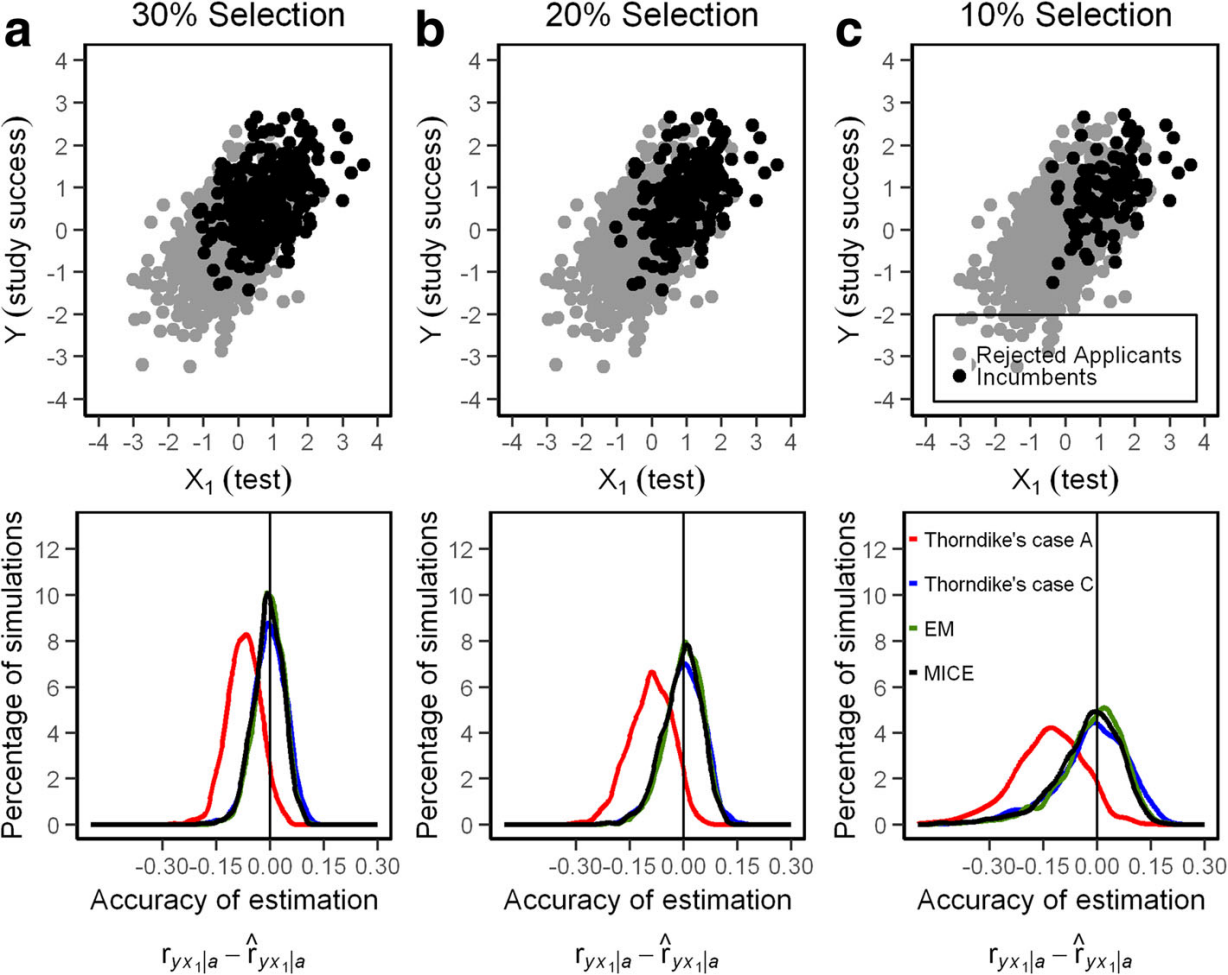
**a**-**c** Scattergram of Y (study success) with X_1_ (test results) and precision of the estimation of ${r}_{yx_1\mid a}$ (predictive validity) from different methods when 30%, 20%, and 10% of applicants are selected

The modal value of all correction methods (${\widehat{r}}_{yx_1\mid a}\Big)$, except for Thorndike’s case A, is fairly close to the target value of ${r}_{y{x}_1\mid a}$ (Fig. 4a-c). Table 1 also shows the root mean square error (RMSE). RMSE is the standard deviation of the prediction error. It shows how close the recovered validity coefficients are to the true validity. Lower values of RMSE indicate better fit.

**Table 1:** Mean of precision of the estimation of ${\mathrm{r}}_{\mathrm{y}{\mathrm{x}}_1\mid \mathrm{a}}$ (predictive validity) from different methods when 30%, 20%, and 10% of applicants are selected with RMSE and confidence interval

	30% Selection				20% Selection				10% Selection			
	mean	SD	RMSE	CI 95%	mean	SD	RMSE	CI 95%	mean	SD	RMSE	CI 95%
Thorndike’s case A	−0.07	.05	.085	−0.16 – 0.02	−0.10	.07	.119	−0.23 – 0.02	−0.14	.10	.167	−0.34 – 0.04
Thorndike’s case C	0.00	.04	.043	−0.09 – 0.07	−0.01	.06	.065	−0.14 – 0.10	−0.02	.10	.102	−0.24 – 0.15
EM	0.00	.04	.039	−0.09 – 0.07	−0.01	.06	.058	−0.13 – 0.10	−0.02	.09	.092	−0.23 – 0.12
MICE	−0.01	.04	.040	−0.09 – 0.07	−0.01	.06	.059	−0.14 – 0.09	−0.03	.09	.097	−0.24 – 0.12

Note. *mean* mean of the accuracy in the 1000 simulations, *RMSE* Root Mean Square Error, *CI* Confidence Interval

Thorndike’s case A formula shows a small bias as the distributions peak left of the true correlation underestimating predictive validity, even when the first order correlation ${r}_{yx_1\mid a}$ was replaced by the semipartial correlation ${r}_{yx_{1\bullet 2}\mid a}$. However, the true correlation lies well within the confidence interval of the 1000 replication runs showing that this bias is rather insignificant. The smallest confidence interval of estimations for ${\widehat{r}}_{y{x}_1\mid a}$is delivered by EM and the largest by the formulas for direct and indirect range restriction. With the simulation problem at hand, MICE did not perform better than EM although MICE is regarded as the most advanced method for coefficients estimation when data are missing [15].

The variation of estimated unrestricted correlations not only depends on the precision of the method, but even more so on the ratio of selection. If the selected group gets smaller (20% in Fig. 4b and 10% in Fig. 4c) the confidence interval grows larger up to a point were usefulness becomes questionable (Table 1).

## Study 2: Application to an entrance test for medical school

In this study we demonstrate a correction for compensatory selection and range restriction with data obtained from the Hamburg Natural Science -Test (HAM-Nat) [27]. The HAM-Nat in combination with hGPA is used for the selection of medical students in Hamburg, Germany. Applicants with hGPA above a certain level are invited to take the test. It consists of 80 MC-Items from biology, chemistry, physics and mathematics covering the scope of high school teaching. The hGPA is used again in combination with HAM-Nat score, to establish the rank order for admission. School grades (hGPA) range from 1.0 (excellent) to 6.0 (insufficient) in Germany. In the year 2011, 207 out of 714 applicants (29%) were admitted by this procedure. As outcome criterion we consider study success as defined by performance in 11 written exams in the first two study years, mainly from biochemistry, physiology, and anatomy, measured as the mean percentile.

### Results

**Fig. 5:**
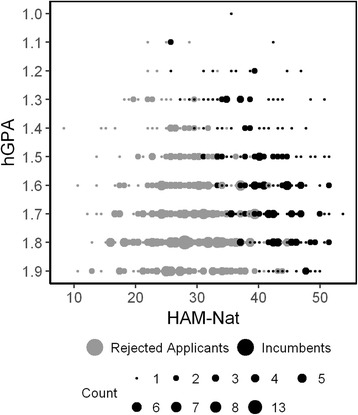
Scattergram of HAM-Nat and hGPA at Hamburg medical school for the 2011 cohort

The compensatory relation between HAM-Nat and hGPA imposes a negative correlation between these predictor variables in the incumbents (Fig. 5) by the same logic that has been demonstrated in the preceding simulation section (Fig. 1). An applicant with a low hGPA can still be accepted if her natural science knowledge test is superior.

We have withheld a complication in our empirical example to keep the description of correction methods as simple as possible. Actually there is a fourth variable which modifies the rank order in half of the accepted applicants according to their performance in a test of social competence [28]. This is not easy to model and we do not expect much change in the estimation of a corrected validity due to this complication. Therefore we bypass it for now. A validity coefficient is not a constant of nature that needs to be known with as many digits as possible. Considering the sizable error that is made by the widespread omission of appropriate corrections, a less than perfect estimation seems tolerable.

Being good at natural science, a student is well equipped to understand the subject matter of the first terms in medical school. But having on average a lower hGPA, which was compensated by her or his high science test score would mean that she or he lacks some of the abilities associated with a high hGPA, presumably abilities which help to negotiate an educational system, dubbed the “academic backbone” by McManus [29]. A less than strong academic backbone would counteract the auspicious prospect good natural science knowledge would bring about.

**Fig. 6:**
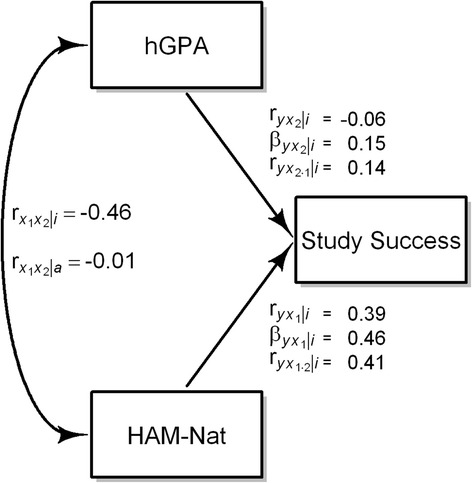
Relations between HAM-Nat, hGPA, and Study Success in the incumbents. ${r}_{y{x}_1}$: first order correlation, ${\beta}_{y{x}_1}$: beta coefficient, ${r}_{yx_{1\bullet 2}}$: semipartial correlation

Due to reciprocal suppression the first order correlation of both variables with the outcome measure is lower than the correlation of the residuals obtained when the linear effect of hGPA is taken out of the HAM-Nat scores and vice versa (Fig. 6). It rises from .39 to .41 for the HAM-Nat and from −.06 to .14 for hGPA.

The ratio of standard deviations for the unrestricted sample relative to the restricted sample is $\mathrm{u}={\mathrm{SD}}_{{\mathrm{x}}_1\mid \mathrm{a}}/{\mathrm{SD}}_{{\mathrm{x}}_1\mid \mathrm{i}}=0.67$ for the HAM-Nat. Different methods of correction for range restriction yield the values given in Table 2 for the predictive validity of the HAM-Nat.

**Table 2:** Estimation results of the validity of the HAM-Nat by four different methods

Correction Method	Validity of HAM-Nat (${\widehat{r}}_{y{x}_1\mid a}$)
Thorndike’s case A	.56
Thorndike’s case C	.59
Expectation maximization (EM)	.60
Multiple Imputation by Chained Equations (MICE)	.59

The estimations from Thorndike’s case C formula, EM and MICE and are close when used for the empirical data from the HAM-Nat (Table 2). Therefore we take the estimation of ${\widehat{r}}_{y{x}_1\mid a}=.59$ as the best estimation of the predictive validity of the HAM-Nat corrected for indirect range restriction. The effects of the two artifacts involved can be decomposed: Correcting for compensatory selection raises ${\widehat{r}}_{y{x}_1\mid a}$ from .39 to .41, and correcting for range restriction raises ${\widehat{r}}_{y{x}_1\mid a}$ further from .41 to .59.

## Discussion

### Approach from classical test theory

An alternative approach to correct for range restriction due to indirect selection is Hunter and Schmidt’s stepwise procedure based on classical test theory [30]. It requires information about the reliabilities of predictor and outcome measures. Different from most approaches not the empirical correlation between Y and X_1_ is estimated, but the operational validity which is defined as the theoretical correlation ${r}_{\rho {x}_1\mid a}$ between the true score of Y, denoted as ρ, and X_1_. The empirical correlation ${r}_{y{x}_1\mid a}$, which according to classical test theory would be ${r}_{y{x}_1\mid a}={r}_{\rho {x}_1\mid a}/\sqrt{\left({r}_{yy\mid a}\right)}$, cannot be recovered by this method, because the reliability of Y in the unrestricted sample, *r*_*yy* ∣ *a*_, is not known.

For the HAM-Nat data presented above the approach by Hunter and Schmidt yields an estimation of ${\widehat{r}}_{\rho {x}_1\mid a}=0.66$ if the reliability of X_1 ∣ a_ = 0.8 and the reliability of Y_∣i_ = 0.7. Obviously the estimation of ${\widehat{r}}_{\rho {x}_1\mid a}$ depends on the reliability estimations for X_1_ and Y, which in turn depend on the method used, e.g. retest-reliability, split half reliability, or some coefficient of internal consistency such as Cronbach’s alpha or omega. As most methods for the estimation of reliability suffer from drawbacks [31, 32] the resulting imperfection is passed on to the estimation of ${\widehat{r}}_{\rho {x}_1\mid a}$. This method leaves us with the operational validity – a true score correlation.

### True score correlation?

Should a correlation coefficient indicating predictive validity be corrected for attenuation by the unreliability of the test scores involved? Few researchers use such corrections, even when they otherwise adhere to classical test theory (CTT), but some advocate them, e.g. McManus et al. reporting operational validity and construct-level predictive validity [3], which is expressed as the correlation of true scores of both, predictor and criterion. In a paper on measurement Schmidt and Hunter make a strong case for using correlations corrected for attenuation instead of empirical correlations because such measures are supposed to be better estimators of the relations commonly sought which would be relations between constructs, not just between test scores [33]. However this direct identification of the statistical expectation of a test (the true score in CTT terminology) with a construct is strongly repudiated by critics like Borsboom and Mellenbergh [34–37]. Simply substituting the term “expectation” by “true score” does not turn the statistical expectation of a test score into a meaningful psychological concept.

The meaning of the true score for the HAM-Nat gets complicated after a little reflection. Why would scientific knowledge predict study success? Certainly because it is auspicious to enter the university with a good foundation for the curriculum of the first two years. But most probably other more basic third factors are in the game which act upon scientific knowledge as well as on study success, factors like motivation, conscientiousness, and intelligence. The knowledge test measures these factors silently by proxy because all of them are needed to achieve a high level of knowledge. If the knowledge measure would be stripped of such associations, how much would be left of its predictive power? Pure knowledge independent of motivation, conscientiousness, and intelligence (which is hard to imagine), would merely consist of the stuff that had been learned devoid of its link to enduring personal dispositions. Knowledge is not an enduring trait such as intelligence, or conscientiousness, it is acquired and, if not used, forgotten over the years. Its causal leverage is confined to the narrow range of the tasks to which it pertains, in this case the natural science content of the medical curriculum.

### A successful test obscures its own validity

Applicants for medical school in Germany are usually well informed about the tests, which are in store for them. They prepare for the test if they judge their chances as sufficiently high, otherwise they neither submit an application, nor spend time for preparation. If competition is strong, and it certainly is with a selection rate of almost 30% in Hamburg in 2011, then the level of preparation required to stand a chance rises and with it the effect of self selection into or out of the pool of applicants. Thus the mere presence of the test exerts a selective force even before the first multiple choice box is checked. This selective force also promotes a high level of ability in the accepted 30%, and if the ceiling of the test is approached its variance is depleted. High level of ability and depletion of variance both work together to reduce whatever correlation might be found between the test and outcome variables reflecting study success. In the end all applicants are well equipped to live up to the natural science demands of the curriculum and no covariance with differential outcome in this respect is left. The success of the test will have erased the possibility to demonstrate its effectiveness.

## Conclusions

Empirical bivariate predictor/outcome correlations cannot be taken as measures of predictive validity if indirect selection and range restriction exist. Correction methods are available and should be used. For practical purposes Thorndike’s case C formula is a relatively straightforward solution to the range restriction problem, provided distributional assumptions are met. With EM and MICE more precision is obtained when distributional requirements are not met, but access to a sophisticated statistical package such as R is needed.

Can the results of our study regarding a compensatory selection decision be transferred to student selection scenarios that use multiple steps with a single criterion each? Imagine that applicants are shortlisted for a cognitive test based on their hGPA, but the following selection decision is based only on their test score. The range restriction on the hGPA is difficult to estimate as we have to deal with a self-selection bias: applicants might not decide to apply if they think that they do not stand a chance. If solely a cognitive test would be used for the final selection decision (without the possibility to compensate a low test score by other means), a stronger range restriction on the test scores would occur. Then correction for compensatory selection would be unnecessary, but correction for range restriction would have a larger effect. Therefore we think that this correction is even more important to determine the predictive validity when an admission test is used as a single criterion.
